# Studying the factors related to refractive error regression after PRK surgery

**DOI:** 10.1186/s12886-018-0879-y

**Published:** 2018-08-14

**Authors:** Mehdi Naderi, Siamak Sabour, Soheila Khodakarim, Farid Daneshgar

**Affiliations:** 1 Department of Clinical Epidemiology, School of Public Health, Safety Promotion and Injury Prevention Research Center, Shahid Beheshti University of Medical Sciences, Chamran Highway, Velenjak, Daneshjoo Blvd, Tehran, I.R, Iran; 2 Department of Ophthalmology, School of Medicine, Kermanshah University of Medical Sciences, Kermanshah, Iran

**Keywords:** Refractive error regression, PRK surgery, Related factors

## Abstract

**Backgtound:**

Photorefractive keratectomy (PRK) is used for a wide range of refractive errors such as low to moderate myopia, hyperopia and astigmatism. While many improvements have been made in laser application and accuracy as well as the modes of corneal flap removal, and although the results are somewhat predictable, regression of refractive errors is still a common complaint among the patients undergoing refractive surgery with Excimer Laser. We aimed to determine related factors of regression following photorefractive keratectomy (PRK) in different types of refractive errors.

**Methods:**

This cross-sectional study included patients who had undergone PRK more than 6 months previously and investigated refractive error regression and related factors. The participants were those who had PRK eye surgery for the first time from 2013 to 2016 using Technolas 217z100. A refraction value of spherical equivalent > 0.75 D after cycloplegic refraction was defined as refractive error regression.

**Results:**

A total of 293 eyes on 150 subjects were studied. The preoperative refractive error of the eyes were as follows: 5.5% were myopic, 1% were hyperopic, 4.8% had astigmatism, 76% had myopic astigmatism and 12.6% had hyperopic astigmatism. Regressed and non-regressed eyes were assessed using the generalized estimating equations for the probabilistic variables of demographic characteristics, topography and eye refraction. The variables of simulated keratometry astigmatism (simK) (OR = 2.8; *p* = 0.04), 5 mm irregularity (OR = 3.56; *p* = 0.01) and sphere value (OR = 1.98; *p* = 0.01) were significantly related to refractive error regression. There was no significant relationship between the regressed and non-regressed eyes of the same person (*p* ≥ 0.05).

**Conclusion:**

There was a positive relationship between the increase of 5 mm irregularity, simK, sphere value before surgery and refractive error regression. Age, sex and type of refraction error of the patient and the expertise of the PRK surgeon could change the general results; therefore, not all cases should be dealt with identically.

## Background

Current advances in refractive surgery have caused dramatic changes in ophthalmology. Excimer laser photorefractive keratectomy (PRK) is accepted as an effective and desirable method of treating refractive error [1, 2]. PRK is used for a range of refractive errors, including low to moderate myopia, hyperopia and astigmatism [2, 3]. While many improvements have been made in laser applications and accuracy as well as the mode of corneal flap removal, regression of refractive error is still a common complaint among patients undergoing refractive surgery with excimer laser. Recent estimates showed that 3.8% to 20.8% of patients require retreatment after myopia correction. Generally, the need for retreatment after surgery with an excimer laser is 6.8% [4–9]. This is why some patients are not satisfied with this type of surgery.

The factors associated with the need for retreatment after LASIK surgery include a small optical zone [10–12], flap thickness [13], high correction [14], keratometry readings [15], significant astigmatism [6, 10, 16], age over 40 years [16]. In PRK, they include the use of Mitomycin [17], refractive correction > − 5.00 D, smaller optical zone (< 6.00 mm) and unstable fixation during laser ablation [18]. It can be said that the stability of PRK is less satisfactory than with LASIK surgery [19, 20]. Determining the factors associated with regression in PRK surgery can help select the best case for this type of surgery and reduce the financial burden of the patient.

The number of studies have comprehensively that have examined the factors relating to regression of refractive error after PRK is insufficient. The current study was carried out to identify the factors associated with refractive error regression in individuals who had undergone PRK because of refractive error. The prevalence of refractive error regression was examined using the variables of sex and age as well as the medical specialty of the surgeon. The relationships between the factors related to refractive error regression in each subgroup were assessed to determine the moderating effects.

## Methods

The study protocol was approved by the Department of Epidemiology, School of Public Health, at Shahid Beheshti University of Medical Sciences and was conducted at Kermanshah University of Medical Sciences. The cross-sectional study was designed to examine the results of PRK at the Lasik Clinic of Imam Khomeini Hospital in Kermanshah province. The medical records of each patient who had undergone PRK for correction of myopia, myopic astigmatism, astigmatism, hyperopia and hyperopic astigmatism were investigated. The procedures had been done by surgeons having different specialties from 2013 to 2016.

A code was employed to prevent identification of the patient for each record. About 2400 patients (roughly 4600 eyes) had undergone PRK with Technolas 217z100 (Bausch & Lomb) over the course of 3 years. The participants were patients who had undergone PRK at least 6 months in the past. Their medical records were randomly selected and they were invited to undergo supplementary examinations. If an individual had not undergone a follow-up examination or there were defects in the data in his/her medical record, another person was randomly selected. This study received ethical approval from the Institutional Review Board of Shahid Beheshti University of Medical Sciences.

### Photorefractive keratectomy surgery

In PRK, the eye is first anesthetized and the cornea is exposed to alcohol 20% for 15 s. A disk of epithelium with a diameter of 8–9 mm is removed with a sponge. The excimer laser then is applied according to the nomogram that the surgeons have prepared with respect to patient age and amount of refraction. The surgeons applies the nomogram in all cases of myopia, then adds 0.25 D to the myopia value to prevent under-correction. The procedure is performed for astigmatism and hyperopia according to the nomogram. After excimer ablation, a sponge soaked with MMC 0.02% is placed on the stroma (according to amount and type of refractive error at 10 s per 1.0 D of correction). The eye is rinsed with balanced saline solution (30 cc) to dilute and remove residual MMC and a bandage contact lens is placed over the cornea.

Postoperatively, all patients receive a topical antibiotic, corticosteroids and artificial tears. The patients are prescribed chloramphenicol 0.5% drops (four times daily for 5 days), betamethasone 0.1% drops (four times a day for 1 week, decreasing to 3 times a day for 3 weeks) and artificial tears (Artelac; Bausch & Lomb) four times a day for 9 weeks. The bandage contact lens is removed when epithelial healing is complete (5 to 7 days postoperative).

### Regression

Cases of regression refers to eyes recording a spherical equivalent cycloplegic refraction of greater than 0.75 D after 6 months upon examination using an auto kerato-refractometer (Topcon 8900; Japan). The information contained in the patients’ preoperative medical records (eye topography variables, eye refraction variables and demographic variables), surgical records (eye movement rate, optical zone size and surgeon specialty) and the post-surgery information, including the cycloplegic refraction value, were included in the data analysis. Eye refraction variables included the type of refractive error, refractive error value and, in case of astigmatism, eye astigmatism axis.

Preoperative refractive error was considered to be myopia, hyperopia, astigmatism, myopic astigmatism and hyperopic astigmatism. The demographic variables chosen were age, sex and level of education (which is indicative of rate of study, computer use and following postoperative medical advice). These were used as quantitative, qualitative-nominal and qualitative-ordinal variables, respectively. An Orbscan IIz device (Bausch & Lomb; USA) was used to draw the thickness, posterior Diff, irregularity (3 and 5 mm) and simK on the topographic map. Orbscan IIz was used to detect corneal irregularities with radii of 3 and 5 mm and the average high and low corneal points as irregularities. To calculate simK, the Orbscan Ilz found the highest and lowest points on two perpendicular axes and converted the difference between them into the unit of power (D). It also calculated the posterior Diff as the distance between the highest point of the cornea and the BFS surface (ideal spherical surface of the cornea). The thickness (of the central part of the cornea) was measured in microns.

The variables in the surgery files were eye movement rate, optical zone size and surgeon specialty. The eye tracker device recorded the changes in eye movement from the steady state during surgery as positive or negative on the coordinate axes. The optical zone is the area on the cornea which is determined based on pupil size and is influenced by the excimer laser. It is classified into groups of 5.5, 6 and 6.5 mm diameters. The surgeon chose one depending on the situation. Surgeon specialty was classified qualitatively-nominally into four groups: ophthalmologists and strabismus, cornea and retina subspecialists. The confounding effect of age and sex was considered, particularly the moderating role of sex, age and surgeon specialty, was dealt with using stratified analysis.

Power and Sample Size Calculation (version 3.1.2) was used to calculate the sample size at a significance level of < 5% and a power of 80% taking into consideration the 60% prevalence of females in the study. To detect an OR value of 2.5 (clinically important) between exposure and refractive error regression after PRK, a total sample size of 195 eyes was required. Given the 25% probability of data loss and non-compliance of the participants to determine the refraction, 50 eyes were added to the sample size in order to preserve the validity of the results. To increase the power of the study, the surgery files of 150 patients were randomly selected and each was invited to undergo another examination. Of the 2400 who underwent PRK surgery, 1535 were women. For this reason, the proportion of males and females was preserved as much as possible.

The variables of irregularity (3 and 5 mm) and posterior Diff were applied quantitatively and continuously (in mm) and simK was applied quantitatively and continuously (in D). The change in eye movement was recorded by the eye tracker device and its absolute value was used in the analysis. Refractive error regression as the main outcome was divided into regressed and non-regressed groups at a cut-off value of 0.75. To determine the effect of an increase in the sphere and astigmatism value before surgery on regression, their refractive errors were considered as absolute values.

**Fig. 1:**
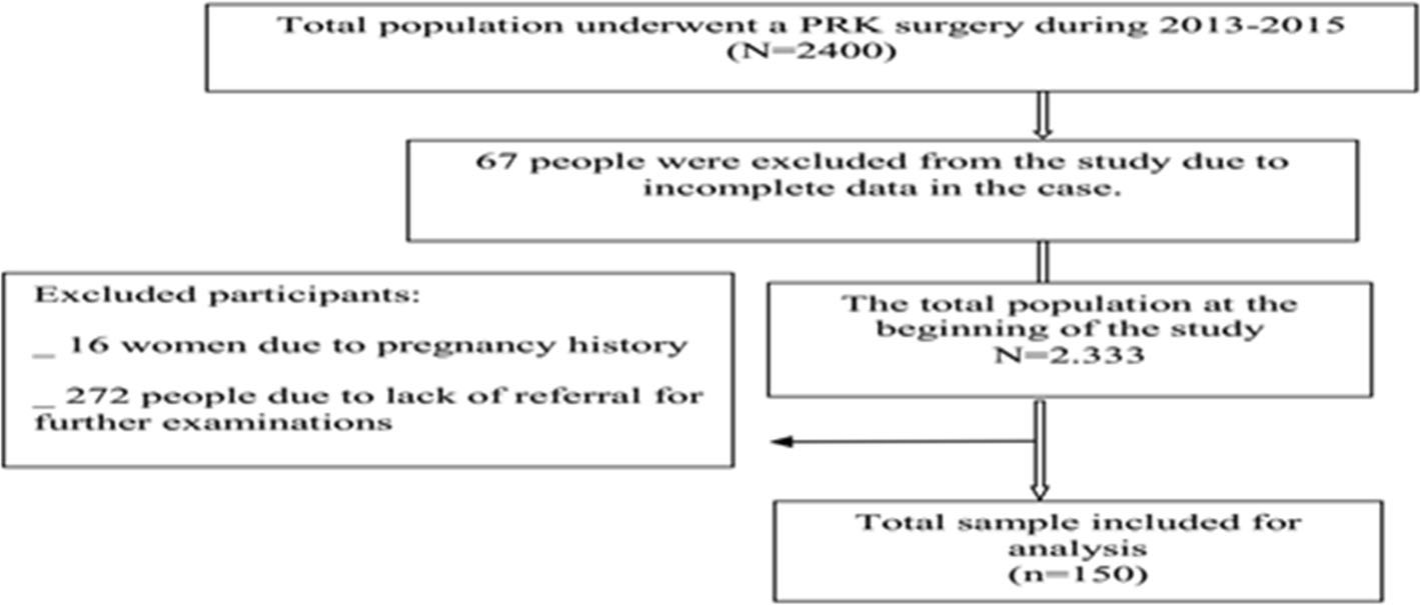
Flow chart of the study population

The preliminary survey assessed about 2400 people. After removal of persons with incomplete case files (67), pregnant women (16) and those who had not been referred for follow-up examinations (272), 2045 eligible people entered the study (Fig. 1). Out of these, 150 were randomly selected.

### Statistical analysis

The statistical analysis was done using SPSS, version 24. Depending on the distribution of the variables, the quantitative variables were described as mean, standard deviation, median, range of change. Qualitative and ordinal variables were described as numerical values and related percentages. The variables of thickness, 3 and 5 mm irregularity, simK, posterior Diff and age were assessed as regressed or non-regressed with regard to the sample size and variable distribution using Mann-Whitney test and t-test. Generalized estimating equations (GEEs) were used to determine the neighbor effect of refractive error regression between the right and left eyes and assess the relationship between all variables and refractive error regression. The moderating role of sex, type of refractive error, age group and surgeon specialty were assessed using stratified analysis.

## Results

The participants totaled 150 patients representing 293 eyes. Seven patients were assessed for one eye each and the remaining 143 had both eyes assessed (286). The participants were 61% female and 39% male. Using the definition of refractive error regression, 56 eyes demonstrated regression. The regression frequency was about 19%. Of the 56 regressed eyes, 16 belonged to 16 patients (one regressed eye per individual) and 40 eyes belonged to 20 patients (both eyes were regressed). The prevalence of regression was 21.1% among females and 15.9% among males. The prevalence of regression for each specialty was 20.7% among general ophthalmologists, 21.6% among strabismus subspecialists, 14.7% among retina subspecialists and 16.4% cornea subspecialists. The prevalence of regression was 17.4% in the under 30 year age group and 21.4% in the over 30 year age group. The prevalence of regression for myopia was 5.9%, hyperopia was 1.3%, astigmatism was 4.2%, myopic astigmatism was 78.1% and hyperopic astigmatism was 10.5%. The preoperative refractive error in the eyes that had undergone PRK were as follows: 5.5% myopia, 1% hyperopia, 4.8% astigmatism, 76% myopic astigmatism and 12.6% hyperopic astigmatism. The characteristics of the patient in terms of other qualitative variables are shown in (Table 1).

**Table 1:** Preoperative characteristics in 293 eyes that undergo PRK Surgery

Factor	Frequency	Percent
Female	180	61.4
Men	113	38.6
Education degree		
Under diploma	47	16
Diploma	91	31.1
Bachelor	115	39.2
Post graduate	40	13.7
Optical zone		
5.5 (mm)	7	2.1
6.00 (mm)	20	6.8
6.50 (mm)	267	91.1
Refraction type		
Myopia (D)	16	5.5
Hyperopia (D)	3	1
Astigmatism (D)	14	4.8
Myopic astigmatism (D)	223	76.1
Hyperopic astigmatism (D)	37	12.6
Surgeon specialty		
Ophthalmologist	135	46.1
Deviation specialty	51	17.4
Retina specialty	34	11.6
Cornea specialty	73	24.9
Astigmatism category (D)		
Astigmatism category (0–2) D	243	82.9
Astigmatism category (2–4) D	47	16
Astigmatism category (4–6) D	3	1
Sphere category (D)		
Sphere category (0–2) D	93	31.7
Sphere category (2–4) D	110	37.5
Sphere category (4–6) D	50	17.1
Sphere category (6–8) D	22	7.5
Sphere category (8<) D	18	6.1
Total	293	100

*PRK* photorefractive keratectomy, *D* Diopter

The mean age of those who had undergone PRK was 31.4 ± 7.7 years. The characteristics of the subjects in terms of other quantitative variables are shown in Table 2.

**Table 2:** Preoperative characteristics in 293 eye that undergo PRK surgery

Factor	Minimum	Maximum	Mean	Std. deviation
Age (y)	20	60	31.4	7.7
Irregularity 3 (mm)	0.4	2.4	1.02	0.33
Irregularity 5 (mm)	0.80	4.90	1.41	0.44
Diff posterior (mm)	0.01	0.04	0.02	0.01
SimK (D)	0.1	3.8	1.2	0.7
Thickness (μm)	453	610	543.6	32.8
Eye movement (mm)	0	24	3.9	2.9
Sphere value (D)	0	12	3.4	2.3
Astigmatism value (D)	0	4.75	1.3	0.94

*PRK* Photorefractive keratectomy, Diff posterior: steepest posterior point difference from Best fit sphere, simulated k astigmatism: keratometry astigmatism, *D* Diopter, *y* year

The comparison between the variables in both the regressed and non-regressed groups showed a significant difference in the mean 3 mm irregularity ($\overline{x}$= 1.16 vs. $\overline{x}$= 0.99, *p* = .001), 5 mm irregularity ($\overline{x}$= 1.61 vs. $\overline{x}$= 1.36, *p* = 0.001) and simK ($\overline{x}$= 1.69 vs. $\overline{x}$ = 1.06, *p* = 0.001) (Table 3). In the eyes with astigmatism, there was no significant relationship between refractive error with type of astigmatism axis (WTR, oblique and ATR; *p* > 0.05).

**Table 3:** Comparison of preoperative characteristics of the eyes with and without regression

Characteristics	Mean		*P* value
	No regression (*n* = 237)	Regression (*n* = 56)	
Age (y)	31.4	31.8	0.72
Thickness(μm)	535.7	529.7	0.21
Irregularity 3 (mm)	0.99	1.16	0.001
Irregularity 5 (mm)	1.36	1.61	0.001
Diff posterior (mm)	0.02	0.02	0.34
SimK (D)	1.06	1.69	0.001
Eye movement (mm)	3.80	4.56	0.07
Sphere value (D)	3.07	5.17	0.001
Astigmatism value (D)	1.09	2.05	0.001

*PRK* Photorefractive keratectomy, Diff posterior: steepest posterior point difference from Best fit sphere, Sim k astigmatism: *simulated keratometry* astigmatism, *P* value: Independent T test

The astigmatism value, 5 mm irregularity, simK and sphere value in the individuals with both eyes regressed and those with both eyes non-regressed showed no significant difference between medians for the left and right eyes (Table 4). Comparison was made of the variables of astigmatism, thickness, 5 mm irregularity, simK and sphere value in right eyes showing regression after PRK for individuals with non-regressed left eyes and vice versa. The only significant difference was observed between the median 5 mm irregularity in the right eye (2.20 vs. 1.80, *p* = 0.03) and in the left eye (1.60 vs. 1.20, *p* = 0.08) (Table 5).

**Table 4:** Relationship between Refractive error regression after PRK Surgery and preoperative variables in people with both eyes regressed and both eyes non-regressed

Variables	People whose both eyes were non regressed after surgery			People whose both eyes were regressed after surgery		
	Right eye *N* = 107	Left eye *N* = 107	Test	Right eye *N* = 20	Left eye *N* = 20	Test
	Mean ± SD	Mean ± SD	*P* value^§^	Mead(min-max)	Mead(min-max)	*P* value®
Thickness (μm)	536.7 ± 32.6	537.6 ± 34.7	0.84	537.5 (499–572)	531 (501–575)	0.49
Irregularity5(mm)	1.33 ± 0.31	1.36 ± 0.38	0.55	1.35 (1.00–2.70)	1.25 (0.9–2.6)	0.45
Astigmatism(D)	0.99 ± 0.70	1.07 ± 0.73	0.43	1.62 (0–3.75)	1.87 (0–4.25)	0.43
Sphere (D)	3.17 ± 1.69	3.12 ± 1.70	0.84	6.25 (0.25–12)	7.87 (0–11.50)	0.41
SimK (D)	0.97 ± 0.49	1.04 ± 0.51	0.30	1.25 (0.25–2.90)	1.50 (0.50–2.90)	0.92

*PRK* Photorefractive keratectomy, Sim k astigmatism: simulated keratometry astigmatism, *P* value: ^§^: Independent sample t. test ®; Mann –Whitney U independent sample test

**Table 5:** Relationship between refractive error regression after PRK surgery and preoperative variables in 16 patients who had one regressed and one non-regressed eyes

Variables	People whose right eyes were regressed after surgery but their left eyes were not surgery			People Whose left eyes were regressed after surgery but their right eyes were not		
	Right eye *N* = 7	Left eye N = 7	Test	Right eye *N* = 9	Left eye N = 9	Test
	Mead(minmax)	Mead(minmax)	*P* value^§^	Mead(min-max)	Mead(min-max)	*P* value^§^
Thickness (μm)	526 (466–573)	520 (474–588)	0.94	505 (470–558)	514 (461–562)	0.72
Irregularity 5 (mm)	2.20 (1.70–4.90)	1.80 (1.20–2.10)	0.03	1.20 (1.00–2.30)	1.60 (1.10–2.40)	0.08
Astigmatism (D)	3.00 (1.50–4.75)	2.25 (1.00–3.75)	0.36	1.5 (1.25–2.75)	2.25 (0.50–4.25)	0.14
Sphere (D)	0.50 (0–5.00)	0.50 (0–4.25)	0.79	2.25 (0.25–7.00)	2.00 (0.50–8.25)	0.82
SimK (D)	2.50 (0.6–3.80)	2.7 (0.9–3.00)	0.94	1.30 (0.80–2.10)	1.75 (0.70–2.90)	0.21

*PRK* Photorefractive keratectomy, *Sim k astigmatism* simulated keratometry Astigmatism, *D* Diopter, *P* value: ^§^: Mann –Whitney Uindependent sample test

The GEEs showed that simK (OR = 2.8, *p* = 0.04), 5 mm irregularity (OR = 3.56, *p* = 0.01) and sphere value (OR = 1.98, *p* = 0.01) were significantly related to refractive error regression after considering the potential confounding variables. Refractive error regression was not influenced by the variables of the other eye of the individual and there was no significant relationship between the regressed and non-regressed eyes of one individual (Table 6).

**Table 6:** Generalized Estimating Equations of independent variable of regression after PRK

Factor	OR (%95 C. I)	*P* value
Age (years)	1.01 (0.95 to 1.06)	0.69
Sex (male vs. female)	1.28 (0.29 to 1.91)	0.79
Thickness (μm)	0.99 (0.98 to 1.03)	0.12
Irregularity 5 (mm)	3.56 (1.32 to 9.22)	0.01
SimK (D)	2.80 (1.01 to 8.20)	0.04
Astigmatism value (D)	2.01 (0.98 to 4.92)	0.06
Sphere value (D)	1.98 (1.59 to 2.54)	0.01
Right or Left	1.07 (0.69 to 1.87)	0.80

Adjusted odds ratio: age, sex, thickness, irregularity 5 mm, simulated keratometry astigmatism, Astigmatism value, Sphere value

The results of stratified analysis on the moderating role of sex, age group, type of refractive error and surgeon specialty showed that the regression of refractive error in females was related to the 5 mm irregularity (OR = 4.2, *p* = 0.02) and simK (OR = 3.6, *p* = 0.04). There was a significant negative relationship between thickness and refractive error regression in males (OR = 0.97, *p* = 0.02). The 5 mm irregularity and simK showed a much weaker relational power and was not statistically significant. As the sphere value increased in females and males, a significant relationship with refractive error regression was observed (Table 7).

**Table 7:** Comparing the results of GEE analysis in women and men to examine the role of gender moderating

Factor	Male (*n* = 113)		Female (*n* = 180)	
	*P* value	OR (%95 C. I)	*P* value	OR (%95 C. I)
Age (years)	0.25	1.04 (0.81 to 4.05)	0.71	0.98 (0.90 to 1.06)
Left or right eye	0.75	0.88 (0.30 to 2.35)	0.67	1.18 (0.53 to 2.64)
Thickness (μm)	0.02	0.97 (0.94 to 0.99)	0.66	0.99 (0.98 to 1.03)
Irregularity 5 (mm)	0.09	4.00 (0.85 to 19.48)	0.02	4.14 (1.17 to 14.58)
SimK (D)	0.54	1.71 (0.39 to 10.8)	0.04	3.61 (1.05 to 12.43)
Astigmatism value (D)	0.10	4.78 (0.72 to 33.01)	0.23	1.81 (0.86 to 4.84)
Sphere value (D)	0.01	1.90 (1.39 to 2.60)	0.01	2.30 (1.66 to 3.18)

Odds ratio: age, thickness, irregularity 5 mm, simulated keratometry astigmatism, astigmatism value, sphere value, *D* Diopter

The relationship between the variables and the regression of refractive error was examined for the myopic astigmatism and hyperopic astigmatism groups. It was found that results of the simK and 5 mm irregularity differed between groups (Table 8). The relationship between these variables was also examined by age group. It was found that only the 5 mm irregularity in the under 30 age group (OR = 3.98, *p* = 0.03) had a significant relationship with refractive error regression. In both groups, a significant relationship with refractive error regression was found with an increase in sphere value.

**Table 8:** Comparing the results of GEE analysis in eyes hyperopic astigmatism and myopic astigmatism

Factor	Myopic astigmatism		Hyperopic astigmatism	
	*P* value	OR (%95 C. I)	*P* value	OR (%95 C. I)
Sex (male vs. female)	0.80	1.18 (0.31 to 4.39)	0.65	1.61 (0.19 to 13.41)
Age (years)	0.80	0.98 (0.89 to 1.09)	0.75	0.98 (0.89 to 1.08)
Left or right eye	0.50	0.79 (0.40 to 1.56)	0.96	0.95 (0.15 to 6.09)
Thickness (μm)	0.13	0.98 (0.97 to 1.03)	0.51	0.98 (0.93 to 1.03)
Irregularity 5 (mm)	0.02	4.04 (1.24 to 13.13)	0.74	1.43 (0.16 to 12.98)
SimK (D)	0.02	5.11 (1.26 to 20.58)	0.66	0.69 (0.13 to 3.60)
Astigmatism value (D)	0.58	1.33 (0.48 to 3.59)	0.17	4.37 (0.51 to 37.44)
Sphere value (D)	0.01	2.41 (1.72 to 3.36)	0.01	2.16 (1.51 to 3.11)

Odds ratio: age, thickness, irregularity 5 mm, simulated keratometry astigmatism, astigmatism value, sphere value, *D* Diopter

The refractive error regression versus surgeon specialty was also examined. For ophthalmologists, only the 5 mm irregularity (OR = 2.7, *p* = 0.04) and simK (OR = 7.8, *p* = 0.03) had a significant relationship with refractive error regression. No significant relationship was found between these variables and refractive error regression for strabismus, cornea and retina subspecialists. A positive relationship was observed between the increase in sphere and astigmatism values and regression of refractive error for all specialties.

The relationship between variables was examined according to type of refractive error. For myopic astigmatism, only the 5 mm irregularity (OR = 5.11, *p* = 0.02) and simK (OR = 4.4, *p* = 0.02) had significant relationships with refractive error regression. For hyperopic astigmatism, no significant relationship was found between these variables and refractive error regression. In both types, a significant relationship was observed between an increase in the probability of refractive error regression and an increase in sphere value.

## Discussion

The current study found a relationship between simK and 5 mm irregularity and refractive error regression after PRK. An increase in these variables increased the likelihood of refractive error regression after PRK. These results emphasize that the overall results cannot be generalized to all patients. For example, unlike for females, no males showed a significant relationship between the 5 mm irregularity and simK and refractive error regression. Although the relational power of thickness and regression was stronger in males than in females, there was a significant negative relationship between thickness and refractive error regression. In the assessment of factors related to refractive error regression versus age group, the 5 mm irregularity was the only variable that had a positive and significant relationship with regression. This underlines why the age, sex and type of refractive error of the individual are important during decision-making.

In addition, the specialty of the surgeon performing PRK showed a relationship with the regression of refractive error. Only general ophthalmologists showed an effect for simK and 5 mm irregularity. The other subspecialties showed no effect. As in previous studies, there was a positive relationship between an increase in sphere value and astigmatism. An increase in refractive error increased the likelihood of refractive error regression [6, 13, 18].

No significant relationship was found between refractive error regression and education level, type of refractive error, surgeon specialty, type of astigmatism axis or posterior Diff. Studies also have reported no significant relationship between age, sex and the thickness and regression of refractive error after PRK [6, 18], although some studies have shown age as a risk factor for regression of refractive error after LASIK surgery [13]. In the present study, although the surgeon specialty showed no significant association, it could be said that surgical decisions such as patient selection, surgical technique and nomogram selection for PRK is very important [6, 21].

No relationship was found between the rate of eye movement and regression of refractive error. These results are inconsistent with the findings of Mohammadi et al. [18] The reason for this discrepancy in the results may be that, in Mohammadi et al., the eye movement rate was qualitatively affected by the surgeon observation and view, but the present study used the range of change (the eye movement rate as recorded by the eye tracking device).

As in previous studies, no relationship was found between the optical zone and refractive error regression [21, 22]. This is inconsistent with the results of Mohammadi et al [18] As in Pokroy et al., the present study observed a relationship between the preoperative refractive error high and the regression of the refractive error. The greater the preoperative refractive error, the greater the likelihood of refractive error regression [10].

Although PRK is safe and effective, it is usually recommended for patients with mild to moderate refractive error [23–25]. This study was focused on the factors related to refractive error regression after PRK and had some weaknesses in terms of the use of variables indicating refractive error regression. One weakness was the lack of assessment of the relationship between epithelium tissue repair disorder and hyperplasia of epithelium tissue in refractive error regression after PRK [26–28].

Because of insufficient information about the amount and administration of medication as well as dryness of the eyes after surgery [29, 30], it was not possible investigate the relationship between these variables and refractive error regression after PRK. Technological advances in corneal imaging have made the precise measurement of anterior and posterior corneal curvature and corneal thickness possible [31]. The careful use of this data can help in the planning of refractive surgery such as PRK.

Insufficient information about employment, history of eye infection and visual activities such as computer use and reading time per day prevented investigation of a relationship between these variables and refractive error regression after PRK. The similarities between the study population and the population targeted by PRK in other communities in terms of gender and age groups mean that the results could be generalized to all except pregnant women, who were excluded from the study.

## Conclusion

The current study showed that, in general, a relationship exists between the variables of 5 mm irregularity, simK and sphere value before surgery and the regression of refractive error; however, the variables of age, sex and type of refractive error and surgeon specialty could change the general results. Therefore, not all individuals should be treated alike. It is recommended that general ophthalmologists consider these variables before intervention in order to determine the best candidates for PRK. It is recommended that insurance companies takes measures to prevent the waste of financial resources on candidates for PRK with a high risk of post-surgery refractive error regression. In addition, the health system should propose a protocol for advising candidates of PRK based on age, sex and other characteristics to the type of surgery that will minimize the likelihood of regression.

## Abbreviations

CI: Confidence interval
D: Diopters
Diff posterior: steepest posterior point difference from Best fit sphere
OR: Odds ratio
PRK: Photorefractive keratometry
SD: Standard deviation
SE: Spherical equivalent
SimK: Simulated keratometry astigmatism
y: year

## References

[1] Lee JB, Choe CM, Seong GJ, Gong HY, Kim EK (2002). Laser subepithelial Keratomileusis for low to moderate myopia: 6-month follow-up. Jpn J Ophthalmol.

[2] Liu YL, Tseng CC, Lin CP (2017). Visual performance after excimer laser photorefractive keratectomy for high myopia. Taiwan J Ophthalmol.

[3] Sher NA, Barak M, Daya S, DeMarchi J, Tucci A, Hardten DR, Frantz JM, Eiferman RA, Parker P, Telfair WB (1992). Excimer laser photorefractive keratectomy in high myopia. Arch Ophthalmol.

[4] Shojaei A, Mohammad-Rabei H, Eslani M, Elahi B, Noorizadeh F (2009). Long-term evaluation of complications and results of photorefractive keratectomy in myopia: an 8-year follow-up. Cornea.

[5] Vaddavalli PK, Yoo SH, Diakonis VF, Canto AP, Shah NV, Haddock LJ, Feuer WJ, Culbertson WW (2013). Femtosecond laser-assisted retreatment for residual refractive errors after laser assisted in situ keratomileusis. J Cataract Refract Surg.

[6] Randleman JB, White AJ, Lynn MJ, Hu MH, Stulting RD (2009). Incidence, outcomes, and risk factors for retreatment after wavefront-optimized ablations with PRK and LASIK. J Refract Surg.

[7] Yuen LH, Chan WK, Koh J, Mehta JS, Tan DT, SingLasik Research Group (2010). A 10-year prospective audit of LASIK outcomes for myopia in 37,932 eyes at a single institution in Asia. Ophtalmology.

[8] Wagoner MD, Wickard JC, Wandling GR, Milder LC, Rauen MP, Kitzmann AS, Sutphin JE, Goins KM (2011). Initial resident refractive surgical experience: outcomes of PRK and LASIK for myopia. J Refract Surg.

[9] D'Arcy FM, Kirwan C, O'keefe M (2012). Ten year follow up of laser in situ keratomileusis for all levels of myopia. Acta ophtalmol.

[10] Pokroy R, Mimouni M, Sela T, Munzer G, Kaiserman I (2016). Myopic laser in situ keratomileusis retreatment: incidence and associations. J Cataract Refract Surg.

[11] Chen YI, Chien KL, Wang IJ, Yen AM, Chen LS, Lin PJ, Chen TH (2007). An interval-censored model for predicting myopic regression after laser in situ keratomileusis. Invest Ophthalmol Vis Sci.

[12] Lian J, Zhang Q, Ye W, Zhou D, Wang K (2002). An analysis of regression afterlaser in situ keratomileusis for treatment of myopia. Zhonghua Yan Ke Za Zhi.

[13] Flanagan GW, Binder PS (2006). Role of flap thickness in laser in situ keratomileusis enhancement for refractive undercorrection. J Cataract Refract Surg.

[14] Gazieva L, Beer MH, Nielsen K, Hjortdal J (2011). A retrospective comparison of efficacy and safety of 680 consecutive LASIK treatments for high myopia performed with two generations of flying-spot excimer lasers. Acta ophtalmol.

[15] Christiansen SM, Neuffer MC, Sikder S, Semnani RT, Moshirfar M (2012). The effect of preoperative keratometry on visual outcomes after moderate myopic LASIK. Clin Ophthalmol.

[16] Kruh JN, Garrett KA, Huntington B, Robinson S, Melki SA (2017). Risk factors for retreatment following myopic LASIK with femtosecond laser and custom ablation for the treatment of myopia. Semin Ophthalmol.

[17] Sy ME, Zhang L, Yeroushalmi A, Huang D, Hamilton DR (2014). Effect of mitomycin-C on the variance in refractive outcomes after photorefractive keratectomy. J Cataract Refract Surg.

[18] Mohammadi SF, Nabovati P, Mirzajani A, Ashrafi E, Vakilian B (2015). Risk factors of regression and undercorrection in photorefractive keratectomy. Int J Ophthamol.

[19] Dirani M, Couper T, Yau J, Ang EK, Islam FM, Snibson GR, Vajpayee RB, Baird PN (2010). Long-term refractive outcomes and stability after excimer laser surgery for myopia. J Cataract Refract Surg.

[20] Hashemi H, Ghaffari R, Miraftab M, Asgari S (2016). Femtosecond laser-assisted LASIK versus PRK for high myopia: comparison of 18-month visual acuity and quality. int ophtalmol.

[21] Pokroy R, Mimouni M, Sela T, Munzer G, Kaiserman I (2017). Predictors of myopic photorefractive keratectomy retreatment. J Cataract Refract Surg.

[22] Frings A, Richard G, Steinberg J, Druchkiv V, Linke SJ, Katz T (2016). LASIK and PRK in hyperopic astigmatic eyes: is early retreatment advisable. Clin Ophthalmol.

[23] Bricola G, Scotto R, Mete M, Cerruti S, Traverso CE (2009). A 14-year follow-up of photorefractive keratectomy. j Refract surg.

[24] Goes FJ (1996). Photorefractive keratectomy for myopia of −8.00 to-24.00 diopters. J Refract Surg.

[25] Guerin MB, Darcy F, O'Connor J, O'Keeffe M (2012). Excimer laser photorefractive keratectomy for low to moderate myopia using a 5.0 mm treatment zone and no transitional zone: 16-year follow-up. J Cataract Refract Surg.

[26] Gauthier CA, Holden BA, Epstein D, Tengroth B, Fagerholm P, Hamberg-Nyström H (1996). Role of epithelial hyperplasia in regression following photorefractive keratectomy. Br J Ophthalmol.

[27] Ramirez-Florez S, Maurice DM (1996). Inflammatory cells, refractive regression, and haze after excimer laser PRK. J Refract Surg.

[28] Kim TI, Tchah H, Lee SA, Sung K, Cho BJ, Kook MS (2003). Apoptosis in Keratocytes caused by Mitomycin C. Invest Ophthalmol Vis Sci.

[29] Bower KS, Sia RK, Ryan DS, Mines MJ, Dartt DA (2015). Chronic dry eye in photorefractive keratectomy and laser in situ keratomileusis: manifestations, incidence, and predictive factors. J Cataract Refract Surg.

[30] Kymionis GD, Tsiklis NS, Ginis H, Diakonis VF, Pallikaris I (2006). Dry eye after photorefractive keratectomy with adjuvant mitomycin C. J Refract Surg.

[31] Fan R, Chan TC, Prakash G, Jhanji V (2018). Applications of corneal topography and tomography: a review. Clin Exp Ophthalmol.

